# 1031. Can We Safely Reduce Unnecessary Blood Cultures in Patients with Suspected Infection in the Emergency Department? The PredictED Study to Develop and Validate a Bacteremia Prediction Model

**DOI:** 10.1093/ofid/ofad500.062

**Published:** 2023-11-27

**Authors:** Anna Kaal, Soufian Meziyerh, Martijn Dane, Nikki Kolfschoten, Ewout W Steyerberg, Cees van Nieuwkoop

**Affiliations:** Haga Teaching Hospital, The Hague, Zuid-Holland, Netherlands; Leiden University Medical Center, Leiden, Zuid-Holland, Netherlands; Hagaziekenhuis, Den Haag, Zuid-Holland, Netherlands; Hagaziekenhuis Den Haag, Den Haag, Zuid-Holland, Netherlands; Leiden University Medical Center, Leiden, Zuid-Holland, Netherlands; Haga Teaching Hospital, The Hague, Zuid-Holland, Netherlands

## Abstract

**Background:**

Blood cultures are commonly used at emergency departments (EDs), while only 5-10% yields a relevant pathogen. We aimed to develop an automatable prediction model in ED patients with suspected bacteremia. This may reduce the use of blood cultures and prevent potential harm from false-positive blood cultures, while minimizing the risk of missing positive cultures.

**Methods:**

In this observational study, we included consecutive adult patients who had a blood culture taken at the ED of the Haga Teaching Hospital, the Netherlands, for a one-year period. Demographics, laboratory, and outcome data were collected from electronic patient records. We defined 23 candidate predictors for our “full model”, of which nine were used for the "basic" model because they are readily automatable (Table 1). Regression analysis with the Least Absolute Shrinkage and Selection Operator was used to define the model. Bootstrapping was performed for internal validation, including imputation of missing values. We assessed discriminative performance using the C-statistic and calibration using the calibration intercept and slope. Clinical utility was assessed by sensitivity, specificity, negative and positive predictive values and decision curves.

**Figure:**
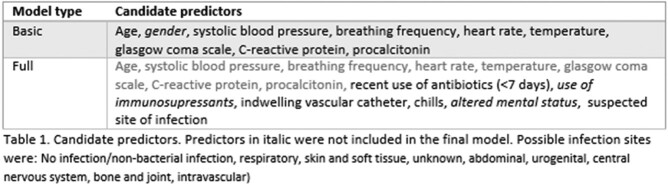

**Results:**

We included 2111 unique patients; mean age 63 years and 46% male. 272 patients had true positive blood cultures (13%); 79 patients (3.7%) had contaminated blood cultures. Our basic model included 8 predictors: age, systolic blood pressure, heart rate, breathing frequency, temperature, Glasgow coma score, C-reactive protein, procalcitonin. The corrected C-statistic was 0.81 (95% CI 0.79-0.83). The full model additionally included recent antibiotic use, indwelling vascular catheter, chills, and suspected infection site. The corrected C-statistic was 0.87 (95% CI 0.85-0.88). Calibration was good for both models. Sensitivity for bacteremia was 97.4% in the basic model, saving 18% of blood cultures (Table 2). The full model could save 26% of blood cultures while maintaining a sensitivity of 98.5% (Table 3).

**Figure:**
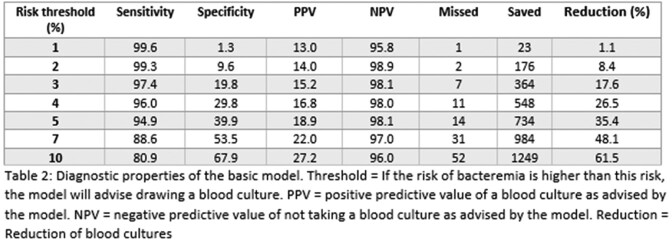

**Figure:**
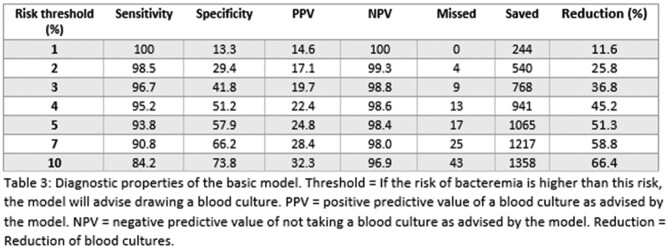

**Figure 1.**
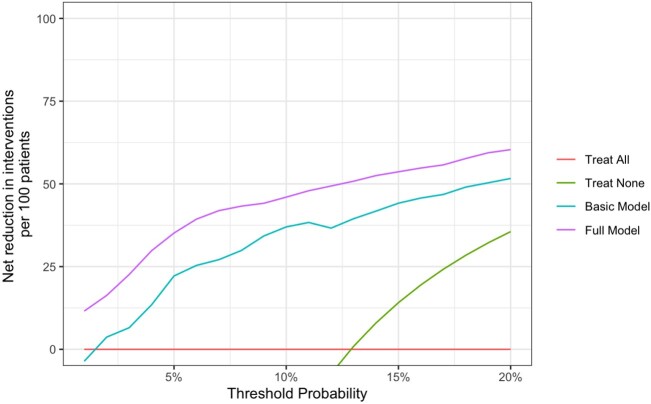
Decision curve analysis

**Conclusion:**

The proposed bacteremia prediction models are designed for easy implementation in electronic patient records and can reduce unnecessary blood cultures. Further external validation is needed before implementation in clinical practice.

**Disclosures:**

**All Authors**: No reported disclosures

